# Histological and ultrastructural aspects of the tongue in undernourished rats

**DOI:** 10.1016/S1808-8694(15)30999-X

**Published:** 2015-10-19

**Authors:** Marilda Aparecida Milanez Morgado de Abreu, Luc Louis Maurice Weckx, Cleonice Hitomi Watashi Hirata

**Affiliations:** a Master's degree, graduate studies at the Stomatology Sector, Otorhinolaryngology and Disturbances of Human Communication Department and the Dermatology Department; Sao Paulo Federal University/Paulista Medical School; b Lecturer, Full Professor of the Pediatric Otorhinolaryngology and Disturbances of Human Communication Department; Hea of the Otorhinolaryngology and Head & Neck Surgery Department, Sao Paulo Federal University/Paulista Medical School; c Master's, Doctor, Head of the Stomatology Sector, Otorhinolaryngology and Disturbances of Human Communication Department and the Dermatology Department; Sao Paulo Federal University/Paulista Medical School. Sao Paulo Federal University/Paulista Medical School (UNIFESP/EPM)

**Keywords:** protein-energy malnutrition, histology, tongue, scanning electron microscopy

## Abstract

There are few published studies on the effects of protein-caloric undernourishment on the oral mucosa.

**Objectives:**

The objective of this study is to verify the histological and ultrastructural aspects of the tongue mucosa in protein-caloric undernourished adult rats.

**Material and Methods:**

A clinical experimental study was done in thirty Wistar rats, 15 controls and 15 with protein-caloric undernourishment. The last group received ration in small amounts, with a reduced casein content, during 45 days. Rats were weighed every 3 days, from the first (90 days of life) to the last day of a 45-day dietary period, when they were sacrificed. Plasma was used for protein electrophoresis and their tongues were prepared for light and scanning electron microscopy. Analysus of variance and Student's t-test were used for statistical analysis.

**Results:**

A significant decrease in weight and in plasma proteins was found in protein-caloric undernourished rats compared to the control group. Histological findings revealed no differences between the two groups and there were no statistically significant differences in the filiform papilla count under the scanning electron microscopy.

**Conclusion:**

Protein-caloric undernourishment does not cause alterations in the tongue mucosa of adult rats.

## INTRODUCTION

Although clinical findings in the mouth such as tongue mucosa atrophy, loss of papillae, and angular cheilitis, are frequently related to nutrition, there are few studies on the effect of protein-energy malnutrition over the oral mucosa and the tongue^1^, ^2^, ^3^, ^4^.

Winkler and Nakamoto in 1982^5^ studied the effects of prenatal malnutrition on tissue development in the mouth of newborn rats. They described histological alterations on the tongue such as reduced cell numbers and the increased size of these cells. Malnutrition also affected incisor and molar dental bacteria. Results suggested that adequate protein intake was extremely important for the healthy development of teeth and the tongue.

Aldred et al. in 1989^6^ reported an increase in yeasts in the mouth of malnourished children, but found no other clear clinical signs.

The aim of this paper is to study the effect of protein-energy malnutrition on the mucosa of the tongue of adult rats, using optical and scanning electron microscopy.

## MATERIAL AND METHODS

This is an experimental study made on thirty “WISTAR” rats aged 90 days and weighing between 180 and 220 grams. Animals were placed in individual cages measuring 40 × 20 × 13 cm each, containing sawdust and water with no restrictions.

Animals were randomly divided into two groups each containing 15 rats. The first group (control) received ration composed of casein (18%), corn (4%), salt (4%), multivitamins (1%), benzoate (0.1%), oil (8%), sugar (10%), corn starch excipient qs (100%). The second group (with protein-energy malnutrition) received a similar ration but with changes in the relative components of casein (1%), given as ration weighing 6 grams/day.

Rats were weighed every 3 days from the first day (age 90 days) until the final day of the 45-day diet period. At this point, having been weighed, the animals were guillotined. Blood was collected and plasma was obtained by centrifugation for protein electrophoresis.

The tongue was sectioned and completely removed. The anterior region of the tongue was sectioned for light and electron microscopy.

Fragments were included in paraffin for microtome sectioning and hematoxylin-eosin (HE) stained. Slides were studied under a common optical microscopy at 50 and 400 times amplification. The following parameters were assessed: epithelial layer uniformity or lack thereof, the presence or absence of an inflammatory infiltrate in the lamina propria, and areas with or without filiform and fungiform papillae in the anterior region of the tongue. Uniformity was considered as the regular distribution of layers with similar cell maturity.

Seven cases in each group (control and malnourished) were assessed by electron microscopy. The number of filiform papillae was one of the study parameters; counts were obtained from electron microscopy pictures taken using VP 120 black-and-white film. Inclusion criteria were: the number of whole filiform papillae (which included a base and a conical projection) counted on pictures magnified 100 times, measuring 9 × 9 cm, using a 2.3 cm^2^ area, in each case counting 5 to 10 regions. Tangential papillae in the left and lower borders were included; tangential papillae in the right and upper borders were excluded; regions with fungiform papillae were excluded^7^.

Statistical analysis included the analysis of variance to compare average rat weight, protein electrophoresis and the papillary count. Student's T-test was used to study the difference between two independent samples^8^ with a 0.05 or 0.5% significance level.

## RESULTS

**Chart 1:**
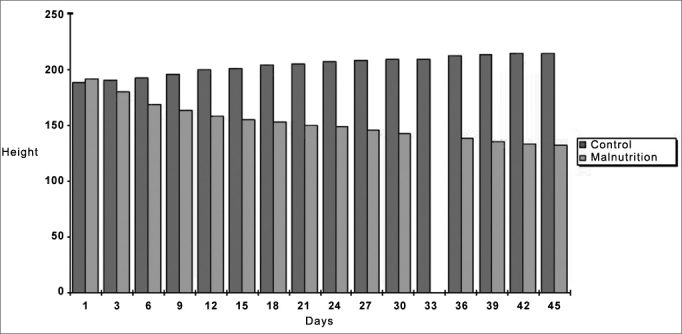
Histogram of rat body weight averages in the control group and the protein-energy malnutrition during the 45-day period.

Weight increased in the control group and decreased in the protein-energy malnutrition group after 45 days. Statistical analysis revealed a significant difference between both samples on the 45th day, as shown on Chart 1.

The plasma protein electrophoresis profile disclosed a significant reduction in total plasma proteins in the protein-energy malnutrition group on the 45th day compared to controls (Tables 1 and 2).

**Table 1:** Results of plasma protein electrophoresis in rats for the control group and the malnutrition group on the 45^th^ day (metrics are total proteins in densitometric units).

Case	Control group	Protein-energy malnutrition group
1	280	210
2	310	200
3	310	215
4	270	200
5	300	230
6	270	205
7	320	200
8	288	215
9	276	220
10	337	230
11	327	200
12	300	220
13	317	259
14	280	260
15	326	250
Average	301	221
Standard deviation	22	20

**Table 2:** Analysis of variance of densitometry results on total electrophoresis in the control group and the protein-energy malnutrition group rats.

Sources of variation	Degrees of freedom	Test		
		T calc		T crit.
Protein-energy malnutrition group rats	14	10,04	(*)	2,05
Control group rats	14			
	28			

**Figure 1:**
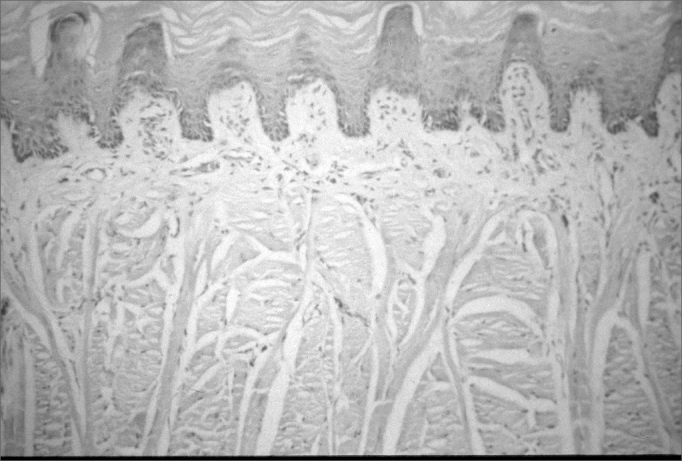
Photomicrograph of a sagittal section of a filiform papilla. Stratified epithelium of the normal tongue mucosa in the control group - Magnification - 100X.

**Figure 2:**
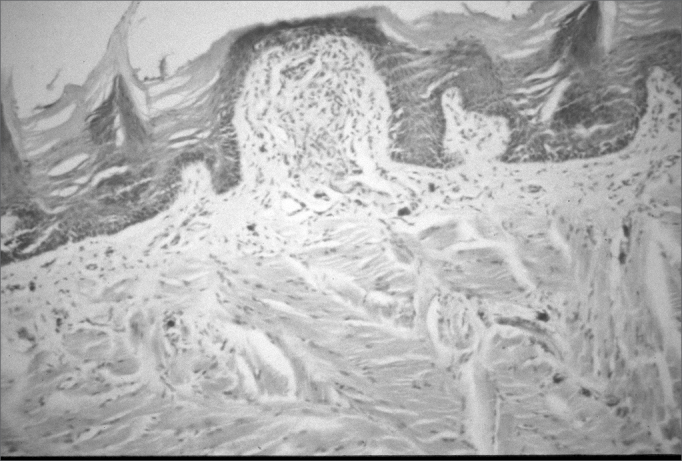
Photomicrograph of a sagittal section of a filiform papilla. Stratified epithelium of the normal tongue mucosa of rats with protein-energy malnutrition - Magnification - 100X.

Common light microscopy histology revealed no differences between the control group and the protein-energy malnutrition group for the parameters we studied (Figures 1 and 2).

Common light microscopy histology of the tongues of rats in both groups showed stratified epithelium with uniform layers in 15 cases. Neither group had an inflammatory infiltrate or areas of papillary loss in the tongue. Keratinization of the ventral surface of the tongue was seen in 2 cases in the control group (with thickening of the keratin layer in 1 control group case) and in no case of the protein-energy malnutrition group.

**Figure 3:**
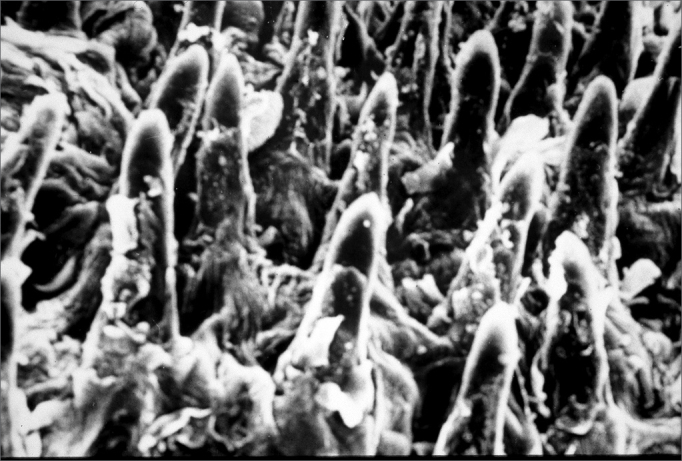
Photomicrograph of the anterior dorsal surface of the adult rat tongue. Filiform and fungiform papillae over which are taste buds. Control group. Original Magnification - 200X.

**Figure 4:**
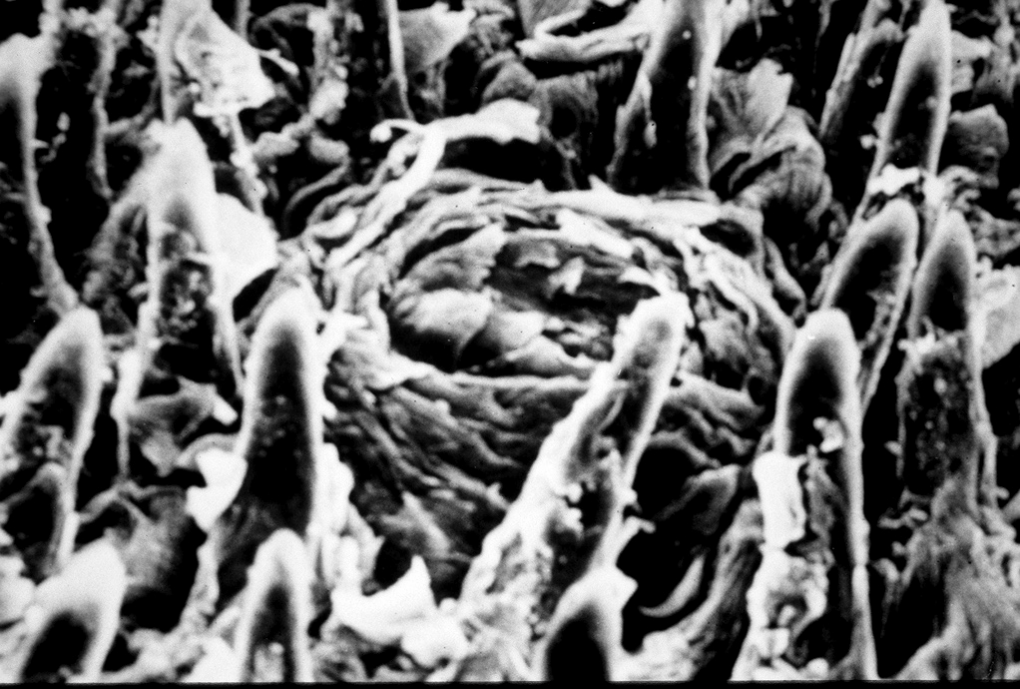
Photomicrograph of the anterior dorsal surface of the adult rat tongue showing a papilla inclined antero-posteriorly. Protein-energy malnutrition group. Original Magnification - 200X.

There were no statistically significant differences in the filiform papillae count by electron microscopy on the tongue in both the control group (7 cases) and the protein-energy malnutrition group (7 cases), as seen on Tables 3, 4 and 5 (Figures 3 and 4).

**Table 3:** Count of filiform papillae on the anterior region of the tongue by unit area (2.3 cm2). Magnification - 100x; control group (electron microscopy).

Control group	Number of papillae per area					
C1	11	13	9	11	9	11
C2	15	14	17	15	16	
C3	18	17	17	17	17	
C4	17	20	20	17	17	
C5	10	10	8	9	10	11
C6	11	13	11	13		
C7	12	11	13	12	12	
Average	13,44					
Standard deviation	3,3					
Sum	484					
Sum of squares	6900

**Table 4:** Count of filiform papillae on the anterior region of the tongue by unit area (2.3 cm2). Magnification - 100x; protein-energy malnutrition group (electron microscopy).

Protein-energy malnutrition group	Number of papillae per area					
D1	17	18	15	19	15	
D2	16	14	10	12		
D3	11	12	14	11		
D4	14	14	15			
D5	13	12	10	11	15	10
D6	11	11	11	14		
D7	11	11	11	12		
Average	13					
Standard deviation	2,41					
Sum	390					
Sum of squares	5244

**Table 5:** Analysis of variance of the number of filiform papillae in the anterior region of the tongue by unit area.

	Degrees of freedom	Test	
		Tcalc	Tcrit
No of papillae in rats with protein-energy malnutrition	30	0.60	2.00
No of papillae in control group rats	36		
Total	66

## DISCUSSION

Malnutrition is a significant public health problem in the world; protein-energy malnutrition is one of the most severe forms of this condition. It affects mostly children in poorer coutries, where the prevalence is estimated as 1/3 of children between birth and five years of age^9^.

The World Health Organization defines protein-energy malnutrition as “the range of pathological conditions arising from a deficiency of cell intake, transport or use of nutrients, commonly associated with infection, occurring with greater frequency in infants and pre-school children”^10^.

Protein-energy malnutrition causes a variety of clinical conditions, the most apparent being effects on body measurements and weight as a whole or for specific organs. Malnutrition develops in a sequence starting from exclusive protein deficiency (kwashiorkor) to protein-energy deficiency (marasmus)^11^. In man, nutritional deficiency is never an exclusively caloric or protein deficiency; both are usually associated.

Clinically there is a strong association between protein-energy malnutrition and hypovitaminosis, and glossities and stomatitis^1^, ^2^, ^3^, ^4^. In out study the 45-day protein-energy deficient diet effectively produced malnutrition in rats aged 90 days, as shown in the statistically significant differences between the control group and the protein-energy malnutrition group; there was both weight loss and a reduction in total proteins. However, the histological study of the tongue of protein-energy malnutrition rats, both under common optical microscopy and electron microscopy did not reveal changes such as glossitis or loss or filiform papillae.

There are no published papers on the effects of protein-energy malnutrition specifically on the mucosa of the mouth or tongue. There is one paper describing changes in prenatal malnutrition^5^. In contrast, there are many reports on the clinical, histological and ultra-structural changes of the tongue mucosa in iron deficiency, particularly during pregnancy and the postnatal period^1^, ^2^, ^3^, ^4^, ^12^, and vitamin deficiency, in particular vitamin B deficiency^12^, ^13^, both of which describe tongue mucosa alterations such as lobulation, fissures, edema and loss of papillae. These studies corroborate our daily clinical findings, where glossitis and loss of papillae are seen in patients with iron deficiency, whereas no evident tongue alterations are seen in patients with protein-energy malnutrition.

Methodological limitations do not allow us to state with certainty that there are no alterations of the tongue mucosa in animals subjected to protein-energy malnutrition, as we studied only adult animals. If our study had included animals in the growing or prenatal phases, which are more sensitive to malnutrition, we might have found some evident change. A further point is the duration of malnutrition. Chronic malnutrition could possibly lead to such changes, as described by some authors^11^, ^14^. Therefore, our investigation opens the door for further studies and new methods.

Using other animal species to understand what happens on the tongue in protein-energy malnutrition leads to a further issue, which is to extrapolate such results to human beings. Although we found no published papers comparing the tongue mucosa between these species, it is known that rats have biological similarities to human beings, which is why it is used so frequently as an experimantal animal^15^.

Regardless of our belief that glossitis is connected to malnutrition and hypovitaminosis in general, we do not exclude the possibility of further reviews on this subject, as other causes are certainly a matter for research. Studies involving prolonged or chronic protein-energy malnutrition, or conducted in prenatal or growing animals, could add further elements to clarify such issues.

## CONCLUSION

Histology and ultra-structural studies of the anterior region of the tongue in 90-day rats subjected to protein-energy malnutrition for 45 days reveal that:

• Protein-energy malnutrition does not alter the tongue mucosa in adult rats.
